# Influence of Aesthetic Appreciation of Wildlife Species on Attitudes towards Their Conservation in Kenyan Agropastoralist Communities

**DOI:** 10.1371/journal.pone.0088842

**Published:** 2014-02-14

**Authors:** Joana Roque de Pinho, Clara Grilo, Randall B. Boone, Kathleen A. Galvin, Jeffrey G. Snodgrass

**Affiliations:** a Natural Resource Ecology Laboratory, Colorado State University, Fort Collins, Colorado, United States of America; b Centro de Administração e Políticas Públicas, Instituto Superior de Ciências Sociais e Políticas, Universidade de Lisboa, Lisbon, Portugal; c Department of Conservation Biology, Estación Biológica de Doñana-Consejo Superior de Investigaciones Científicas, Seville, Spain; d Department of Biology & Centro de Estudos do Ambiente e do Mar, Universidade de Aveiro, Aveiro, Portugal; e Department of Ecosystem Science and Sustainability, Colorado State University, Fort Collins, Colorado, United States of America; f Department of Anthropology, Colorado State University, Fort Collins, Colorado, United States of America; Université de Sherbrooke, Canada

## Abstract

The influence of human aesthetic appreciation of animal species on public attitudes towards their conservation and related decision-making has been studied in industrialized countries but remains underexplored in developing countries. Working in three agropastoralist communities around Amboseli National Park, southern Kenya, we investigated the relative strength of human aesthetic appreciation on local attitudes towards the conservation of wildlife species. Using semi-structured interviewing and free listing (n = 191) as part of a mixed methods approach, we first characterized local aesthetic judgments of wildlife species. With a Generalized Linear Mixed Models (GLMM) approach, we then determined the influence of perceiving four species as beautiful on local support for their protection (“rescuing them”), and of perceiving four other species as ugly on support for their removal from the area, while controlling for informant personal and household socioeconomic attributes. Perceiving giraffe, gazelles and eland as beautiful is the strongest variable explaining support for rescuing them. Ugliness is the strongest variable influencing support for the removal of buffalo, hyena, and elephant (but not lion). Both our qualitative and quantitative results suggest that perceptions of ugly species could become more positive through direct exposure to those species. We propose that protected areas in developing countries facilitate visitation by local residents to increase their familiarity with species they rarely see or most frequently see in conflict with human interests. Since valuing a species for its beauty requires seeing it, protected areas in developing countries should connect the people who live around them with the animals they protect. Our results also show that aesthetic appreciation of biodiversity is not restricted to the industrialized world.

## Introduction

Environmental organizations in industrialized countries have long harnessed the visual and symbolic power of charismatic, “cute” and otherwise visually attractive animals in campaigns garnering public support for conservation causes (e.g., [1]). More recently, understanding the role that human aesthetic appreciation of animal species plays in conservation has become a prominent concern in conservation science. Studies have explored which visual characteristics of animals explain human preferences for them and related attitudes [2], [3], [4], [5], [6], [7], [8], [9]. Others have specifically investigated the influence of animal attractiveness on conservation decision-making, demonstrating that aesthetic judgments of wild animals influence attitudes towards their conservation among the general public [10], [11], [12]. Positive perceptions of animals based on their physical characteristics are also shown to more strongly influence decisions of conservation policy-makers than scientific criteria [13], [14]. Stokes [5] and Marešová and Frynta [3] thus recommend that conservation science pays attention to animal physical attractiveness. For instance, understanding better how the general public and decision makers value species aesthetically can inform conservation strategies of less charismatic species by making them more widely known [5]; counteracting aesthetically-driven biases in species selection for ex-situ conservation [9]; and promoting a more equitable allocation of conservation resources [2], [3], [14], [15], [16], [17], [18] and conservation science funding [19]. Recent outreach initiatives have focused on calling attention to the neglect of “ugly animals” by conservation efforts [20].

How biodiversity is aesthetically valued in developing countries has received much less scholarly attention. Only a few studies have hinted at the aesthetic dimension of human-wildlife relationships in rural Africa [21], [22], [23]. To our knowledge, no work has specifically examined how aesthetic appreciation of animal species influences attitudes towards their conservation among human communities living around protected areas in Africa. Infield [24] and Kuriyan [25] argue that incorporating local non-economic values of wildlife, such as their aesthetic value as we contend, in conservation strategy design can improve their acceptance locally. Others (e.g., [26], [27], [28]) have shown that common ground between conservation objectives and local communities’ goals for species’ management can exist, with the latter not always driven by utilitarian concerns. Instead, local communities have expressed wanting to keep species around for current and future aesthetic enjoyment and cultural reasons [27]. However, as reviewed by Stern [29], the dominant paradigm for explaining relationships between protected areas and neighboring human communities, and for designing conservation policies involving these communities, has been economic rationalism: local residents are presumed by conservationists to respond primarily to conservation-linked economic stimuli.

A case in point is our study area in the Amboseli Ecosystem in southern Kenya. There, tourism revenue-sharing, economic compensation of damages caused by wildlife to livelihoods and community-based conservation initiatives have been choice strategies for promoting local support for wildlife conservation among the pastoralist, agropastoralist and farming communities residing around Amboseli National Park (hereafter, Amboseli NP) [30], [31], [32]. Still, protest killings of charismatic wildlife (e.g., rhinoceros, elephants, lions) by local Maasai pastoralists [33], [34], [35] show that relationships between local communities and the park have remained uneasy despite these efforts. In general, most research on drivers of local attitudes towards wildlife and their conservation in the East African rangelands has explored the influence of people’s demographic and socioeconomic attributes, such as land use, gender, formal education, religious affiliation, and of providing wildlife conservation-based economic benefits to local households. In northern Kenya, a land use type, agropastoralism, is the strongest predictor of negative attitudes towards elephants [21] - a result similar to Okello’s [36] for wild herbivores and carnivores in the Amboseli Ecosystem. Gender is another influential variable: women are more negative than men towards elephants [21] and other species [37]. In Amboseli, Hazzah et al. [40] determined that being an Evangelical Christian is the strongest predictor of negative attitudes towards lions (after losses of livestock to predators). Several studies found a weak effect of formal education on attitudes [21], [37], [38], [39]. In Kenya, wildlife-based economic benefits in households improves local attitudes towards wildlife, although knowledge of the association between conservation and benefits [21] and equitable benefit distribution [38] matter more than the benefits’ value. Other research has addressed how the political economic contexts of relationships between local communities and protected areas affect attitudes and behaviors towards species (e.g., [28]). To our knowledge, no study has examined how characteristics of the animals themselves, e.g., their physical appearance, and people’s perceptions thereof, affect attitudes towards their conservation in this region.

In this article, we address this gap in the conservation literature on human aesthetic appreciation of wildlife in rural Africa. We specifically investigate the influence of perceiving wildlife species as physically attractive (or “beautiful”) on people’s support for their protection; and of perceiving them as physically unattractive (or “ugly”) on support for their removal from the area. Focusing on three Maasai agropastoralist communities located around Amboseli NP in Kajiado County, we first determined which species local residents consider beautiful and ugly, and characterized their aesthetic judgments of these animals. Next, we evaluated the influence of perceiving species as beautiful and ugly on attitudes towards their conservation, i.e., respectively, supporting species’ protection and supporting their removal, while controlling for informant personal and household socioeconomic attributes. Our general hypothesis is that attitudes favoring protection of species and their removal are, at least partly, explained by how people aesthetically judge them – albeit to different degrees across species. We make the case that aesthetic appreciation of species should be investigated in non-industrialized societies, in its own right and because of its potential influence on conservation decision making and conservation design strategy.

## Methods

### Ethics Statement

This research protocol was approved by the Institutional Review Board (IRB) at Colorado State University. All informants agreed to be interviewed. Given their limited literacy level, we could not obtain their written informed consent. We documented oral consent on our interview guides and questionnaires, as approved by Colorado State University’s IRB. In-country research permission was provided through the first author's affiliation with the International Livestock Research Institute (ILRI) in Nairobi. The Imbirikani, Olgulului-Lolarrash and Osilalei Group Ranch Committees granted us local research permission.

### Study Area

The field research was conducted between February 2002 and July 2004 in the semi-arid, wildlife-rich Amboseli Ecosystem. This savanna ecosystem covers about 8,500 km^2^ of eastern Kajiado County [41] in Kenya’s Rift Valley Province and includes the unfenced 392 km^2^ Amboseli NP [42]. One of Kenya’s most visited parks [43], it encloses historic dry season grazing areas for wildlife and local pastoralists’ livestock [44]. In both rainy seasons (March-May; October-December), wildlife disperse out of the park onto surrounding ranches that are privately and communally owned (i.e., group ranches) by Maasai pastoralists and agropastoralists. Western [31] has described Amboseli Maasai, livestock and wildlife as ecologically intertwined and compatible. Historically transhumant herders of cattle and small stock [45], local Maasai land users are now diversifying their economy [46], sending their children to school and becoming Christians [47]. Generally, Maasai do not eat wildlife, except in droughts [48]. Like other East African pastoralists [49], [50], [51], Maasai display a sophisticated appreciation of their cattle’s aesthetic attributes (e.g., coat color patterns; horn shapes) and a related nomenclature [47], [52], [53]. There is, however, only limited work on Maasai aesthetics (see [54]) and none on their aesthetic perceptions of wildlife.

Three decades monitoring the ecosystem’s wildlife populations reveals a complex situation on the Maasai-owned ranches [55]. Imbirikani Group Ranch (hereafter, GR), which includes one of our study sites, shows increases in populations of giraffe (*Giraffa camelopardalis*), Thompson’s gazelle (*Eudorcas thomsonii*), Grant’s gazelle (*Nanger granti*), wildebeest (*Connochaetes* taurinus) and zebra (*Equus burchelli*); and significant declines of impala (*Aepyceros melampus*) and black rhinoceros (*Diceros bicornis*). In a privatized area, a former group ranch sharing a boundary with Imbirikani GR, populations of gazelles, impala, eland (*Tragelaphus oryx*), buffalo (*Syncerus caffer*) and giraffe have significantly decreased. Western et al. [55] attribute these declines to land privatization. In 2008, the ecosystem’s African elephant (*Loxodonta africana*) population was just over 1,500 [56]. Dwindling local populations of the vulnerable African lion (*Panthera leo*) [57] have been ascribed to Maasai lion hunting practices [30], [40].

**Figure 1:**
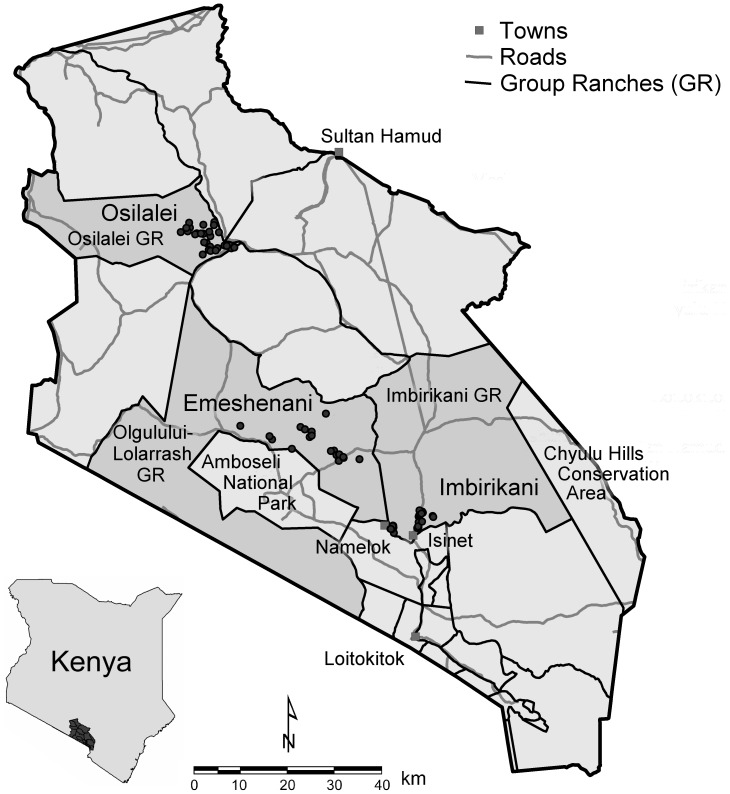
The Osilalei, Emeshenani and Imbirikani study sites within the Greater Amboseli Ecosystem. Dots are the settlements where the interviews took place.

We selected three study sites, Imbirikani, Emeshenani and Osilalei, located at varying distances to Amboseli NP and characterized by contrasting land tenure/use systems and presence/absence of tourism and conservation (Figure 1). Our Imbirikani site, within Imbirikani GR, includes the settlement areas surrounding Isinet and Namelok towns and swamps. Since the 1970s, these areas exhibit a blend of pastoralism and horticulture practiced by both Maasai and non-Maasai farmers. The semi-arid Emeshenani site is located on a ridge at the park’s northern edge in Olgulului-Lolarrash GR. Extensive pastoralism is the main land use. The Osilalei study site, within the former Osilalei GR (subdivided in the 1990’s), is the furthest away from Amboseli NP. Osilalei households combine herding with rainfed cultivation on small private parcels. Residents of the Imbirikani and Emeshenani study sites have access to economic benefits provided by conservation initiatives located on the Imbirikani and Olgulului-Lolarrash GRs. These benefits, provided by the Kenya Wildlife Service and local small-scale community-based conservation initiatives, include employment, health services, secondary education scholarships and outlets for Maasai crafts.

### Data Collection

We followed a mixed-methods approach combining qualitative and quantitative data collection and analyses. Ethnographic data from participant observation (e.g., while herding cattle and visiting protected areas with local residents) and preliminary unstructured interviews with 22 key informants and four focus-groups allowed us to formulate culturally accurate questions, define concepts for the research’s subsequent stages and contextualize and interpret the quantitative results [58]. The data for our statistical analyses came from free lists [59] included in semi-structured interviews [60]. These were conducted across the three study sites with Maasai informants from culturally defined age and gender categories (i.e., elders; married women; *ilmurran* or young men/”warriors”; young unmarried women; boys; girls). In each study site, at least 30 households were randomly selected and, within each, the household head and one dependent were separately interviewed. The final sample of informants (n = 191) includes 109 men (57%) and 82 women (43%). Most of the informants (85.3%) had no formal education (0.82 is the mean number of years in school among those who did) and 60.7% identified themselves as Christians, mostly Evangelical (Table S1).

To determine aesthetic judgments of wildlife species we asked each informant to list the species they found beautiful and the species they found ugly. Free lists like these are simple and powerful tools to qualitatively and quantitatively explore a cultural domain [59], [60] – here, aesthetic preferences for species. Free listing ensured that our analyses focused on the species most relevant to the informants rather than to the researchers (see [61]), which informants listed according to their own aesthetic criteria. We found one Maasai linguistic particularity to be critical: in Maa, the local language, the word *sidai* can be used for both “beautiful” and “good” (also “nice”, “harmless”). To ensure that our data reflected perceived *visual* qualities of animals rather than their perceived likeability, we deliberated on this issue with our key informants and adopted the use of “to please one’s eye”, i.e., *atil* (also “to attract because of beauty”; [52]). Conversely, we used *torrono olkitaunei* (i.e., “of bad formation/appearance”) to convey the notion of physical unattractiveness (“ugliness”) as opposed to animal “badness”, i.e., *atorrono* (“to be bad, evil”). After finishing their lists, informants explained what made each species beautiful and ugly, further helping us apprehend the local concepts of “beauty” and “ugliness”.

In the course of the preliminary focus group and key-informant interviews, we asked our informants to propose management actions for the wild animals they encounter around the park. Their suggestions included killing all of the animals; people being allowed to kill the problematic animals; fencing them in the park; fencing off the agricultural areas; for people and wildlife to stay together as God had created them; and for people to be financially compensated for the losses caused by wildlife. Based on this information, we designed two fictional scenarios that we used in the subsequent semi-structured interviews (n = 191) to explore how aesthetic judgments affect attitudes towards conservation. We asked our informants to list the species they thought pertained to each scenario: 1) “Imagine that the wild animals were disappearing from this land and God gave you the power to rescue some of them, which ones would you rescue?”; and 2) “Imagine that God gave you the power to make some wild animals disappear from this land, which ones would you like to see removed?” Listing species as “to be rescued” was interpreted as reflecting a positive attitude towards their conservation, i.e., support for their protection. Listing species as to be removed was interpreted as reflecting a negative attitude towards the conservation of those species. After finishing each list, informants explained what made each species “to be rescued” and “to be removed”. A few informants did not conduct some listing tasks, leading to variable sample sizes across questions. All interviews were conducted in Maa, translated to English, recorded and transcribed. We also collected data on informants’ personal attributes (i.e., education level; gender; religious affiliation) and their households’ socioeconomic attributes (i.e., land tenure; land use; economic benefits from wildlife in the household) (Table 1).

**Table 1:** Summary of independent variables used in the statistical analysis: aesthetic judgment of species and informant attributes (personal and household) (n = 191).

Variables	Explanation	Legend
**Aesthetic judgment of species**		
Beautiful	Whether informant listed species as beautiful	Not listed as beautiful = 0, listed as beautiful = 1
Ugly	Whether informant listed species as ugly	Not listed as ugly = 0, listed as ugly = 1
**Personal attributes**		
Education	At least some primary education	Uneducated = 0, educated = 1
Gender	Gender	Man = 0, woman = 1
Religion	Religious affiliation	Maasai = 0, Christian = 1
**Household attributes**		
Land tenure	Communal or private land tenure	Group ranch = 0, private ranch = 1
Land use	Pastoralist or agropastoralist	Livestock only = 0, livestock+cultivation = 1
Benefits	Economic benefits from wildlife in the household	No = 0, yes = 1

### Data Analyses

We coded and analyzed informants’ explanations for listing species as beautiful and ugly using NVivo 2, a qualitative analysis software package [62]. Based on our informants’ explanations, we defined animal aesthetic characteristics as including physical attributes (e.g., skin colors/patterns; body shape; size) and how entertaining an animal’s behavior is to viewers. The fact that some informants listed individual species (i.e., Grant’s and Thomson’s gazelles; impala) while others mentioned categories like “gazelles” (*inkoiliin*) presented a coding challenge. Since informants used “gazelles” more frequently, we counted the three species as one generic “gazelle” species. As most informants did not discriminate leopard (*Panthera pardus*) and cheetah (*Acinonyx jubatus*), we also counted them as one “cheetah/leopard” species. These species groupings also make sense because of the species’ common visually distinctive characteristics, as perceived by our informants (i.e., the gazelles’ body shape and colors; the carnivores’ skin spots).

Next, we used Generalized Linear Mixed Models (GLMM) to investigate the effect of perceiving species as beautiful on support for rescuing them while controlling for personal and household socioeconomic variables. We selected for testing the four species that were the most frequently listed as to be rescued (by at least 40 informants), i.e., giraffe, gazelles, eland and zebra. This analysis was based on information-theoretic methods [63]. For each analysis and for each species, we designed a set of 19 candidate models to explain support for rescuing species that were guided by four general hypotheses: 1) perceiving a species as beautiful is the main variable explaining support for rescuing it; 2) personal attributes explain support for rescuing a species; 3) support for rescuing a species is mostly influenced by informant’s household socioeconomic attributes and 4) a combination of each previous hypothesis’ best model explains support for rescuing a species (Tables S2, S3, S4 and S5). We used a logit link function where support for rescuing species (yes; no) is the binomial response variable [63]. We introduced study site as a random effect to avoid pseudo-replication among the three study sites. We ranked the models according to Akaike’s Information Criterion (AIC) [64] and we assessed model accuracy through quantile-quantile plots, examining how well the most supported models (i.e., ΔAIC≤2) fit the data (not shown). We calculated the relative importance of each variable by summing the Akaike weights (W_i_) across the most supported models to estimate the probability that the given variable influences support for rescuing the species. We repeated this analysis for those species that were listed as to be removed by more than 40 informants, i.e., buffalo, elephant, hyena and lion; Tables S6, S7, S8 and S9, respectively). We performed all the statistical analyses with the *lmer* [65], *glmmML* [66] and *ncf* [67] R packages, version 2.10.1. [68]. Finally, to evaluate the role informants ascribe to their own aesthetic perceptions, we qualitatively analyzed the reasons informants gave for rescuing and removing species. To introduce our results below, and throughout the article, we present qualitative quotes that complement and illuminate the statistical results.

## Results

### Beautiful and Ugly Species

**Figure 2:**
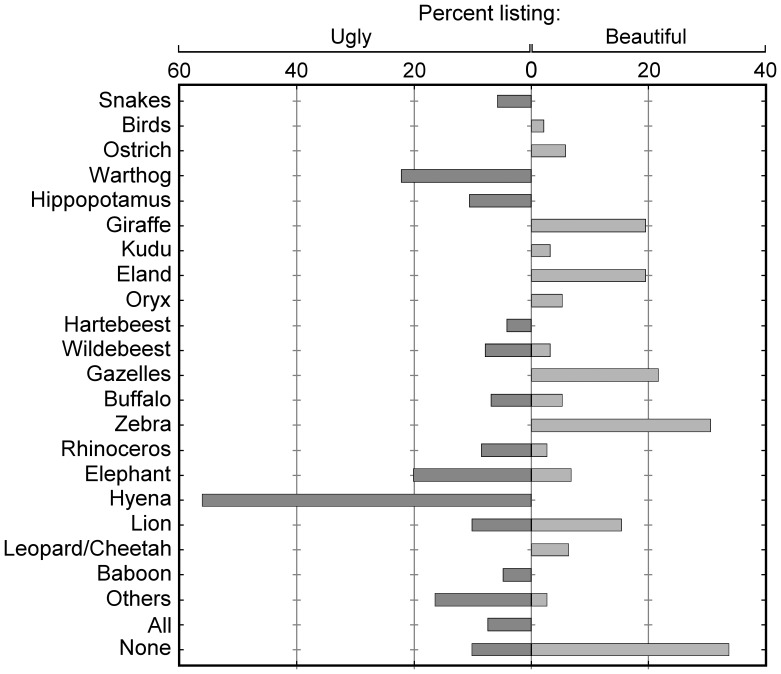
Species listed as beautiful and ugly by informants. Dark gray bars represent percentages of informants who listed each beautiful species (n = 190; multiple species allowed). Light gray bars represent percentages of informants who listed each ugly species (n = 189; multiple species allowed). The “other” category includes species listed less than 1% of the time.

Numerous informants expressed delight at the sight of wildlife on the landscape. For instance, an Emeshenani elder explains: “I was born in a land with many wild animals and it’s beautiful to see them grazing with the cows.” An elderly woman from Imbirikani claims “I like watching them because they are colors put by God on the land. […] They decorate the land.” Seeing wildlife also makes life more exciting: “Without wild animals, we would be bored” says a young unmarried Emeshenani woman. Some informants, however, puzzled by our questions about wildlife’s aesthetic value, listed no wild animal as beautiful (Figure 2). For instance, a young Emeshenani man asks “What beauty in a wild animal?” and an Osilalei woman states that she does not “bother about their beauty and ugliness.” Others were adamant that wildlife cannot be beautiful because they are not cattle (e.g., “Only the cow is beautiful and nicely created”; Married woman, Osilalei).

Confirming this variability in aesthetic judgments, we see that while 66.3% of the 190 informants cited at least one species that “pleases their eye” and listed 19 species in total, some of these beautiful species were perceived as ugly by other informants (i.e., elephant, lion, rhinoceros, wildebeest, buffalo) (Figure 2). The most frequently listed beautiful species were large herbivores, which people praise for their colors and their morphological or behavioral likeness to domestic animals. An Imbirikani elder explains about the eland: “I see it standing and I'd like to cross it with my cow. It’s like a cow and much bigger […] I usually say “Oh! Its color is nice!” I'd like to get one to be my bull. And when elands are eaten by lions, they cry like cows!” A young herder says of the giraffe: “They don’t run away when they see us. They go slowly so we can enjoy looking at them for a long time.” Zebra, especially, are admired for their “perfectly matched black and white stripes” (Married woman, Emeshenani). Notably, some informants characterized lion and elephant as both harmful to humans and livestock *and* beautiful, emphasizing how they offer entertaining, even fascinating, sights: “Everything is different with the lion: how it lies down, walks, stands. Although it kills people, it’s good to look at!” (Elder, Emeshehani). An Emeshenani elder explains how much he enjoys watching them: “I like the lion, even though it’s aggressive. If you see one, you must stop and look at it! […] A lion builds the mind so much! I don’t know what’s really inside, but it must have something magical […] It’s also interesting to watch lions mating” (this quote from [47] was previously published in [26: 341]) As for the elephant, “[It] pleases my eye: I just like to see it taking its hand [trunk] to a tree to eat, calling the other elephants, controlling them; their leader taking them from place to place” (Elder, Imbirikani).

The majority of informants (88.4% of 189) listed at least one ugly species, for 31 species in total (Figure 2). The hyena (*Crocuta crocuta*) is disproportionately listed “because of its colors and the way it’s made. It’s terrible. The way it stands is also very bad. God really didn’t favor it!” (Elder, Emeshenani). Another Emeshenani elder explains: “I could say I’ll go and watch elephants, but I’ll never say I’ll go and watch hyenas!” Other informants find elephants ugly, citing their size, disproportionate teeth and ears, and a generally peculiar appearance. As an Imbirikani elder clarifies: “I don’t even like to look at the elephant. It has no good color and has loose flesh”. When informants listed lions as ugly (which happened less frequently than listing them as beautiful), they mentioned their “bad colors” and “scary appearance”: “If you see a lion from a distance, you notice one color; if you come close and it’s annoyed, you see its color change and its hairs stand up. It's frightening!” (Elder, Imbirikani). Women, particularly, find lions ugly because of their terrifying mane, and the fear lions inspire them in general. Some informants mention the dread that buffaloes, too, trigger: “I don’t know if it’s because they’re dangerous, and so we don’t enjoy looking at them. But I think it’s also how they’re made, with few hairs on their skin” (Elder, Osilalei).

### Relative Effects of Aesthetic Appreciation of Species on Attitudes towards their Conservation

Most informants (77.9% of 190) listed at least one species they would rescue if God gave them the power to do so, totalling17 species, and including gazelles (listed by 45.3%), giraffe (36.3%), zebra (29.5%), eland (25.3%), wildebeest (14.2%), ostrich (*Struthio camelus*; 8.9%), lion (8.4%), oryx (*Oryx beisa;* 5.8%) and elephant (5.3%). Informants gave aesthetic justifications for rescuing these species. For instance, an Imbirikani woman would “rescue zebra, wildebeest, eland, and gazelles because they look beautiful on the land.” Or, says an Emeshenani elder, “I hate the lion, but I’d rescue it because I like to watch it.” An Osilalei elder explained “I’d like to have all the wild animals removed except the ones I said were beautiful” (i.e., buffalo, oryx, zebra and gazelles). An elder in Imbirikani even invoked a locally almost extinct species, the dangerous black rhinoceros (listed by 1.6%): “I'd like for rhinos to come back. Rhinos, elephants, lions and grazers, all are good to look at.”

The informants (n = 184) listed 20 species whose local removal they support, including elephant (42.93%), hyena (42.39%), lion (34.24%), buffalo (28.8%), rhinoceros (17.93%) and wildebeest (16.3%). The danger these species represent and the harm they cause to livelihoods (even wildebeest, by transmitting the deadly malignant catarrhal fever to cattle) was the most frequently cited justification for wishing to remove them. Several informants, though, invoked an animal’s ugliness to explain their negative attitude. For instance, “the hyena, although it’s a cleaner because it eats carcasses and ashes, I want it finished […] because it’s ugly and disturbs people in their sleep” (Elder, Imbirikani). The buffalo should also disappear because “it looks like a fake bull” (Young married woman, Emeshenani).

**Figure 3:**
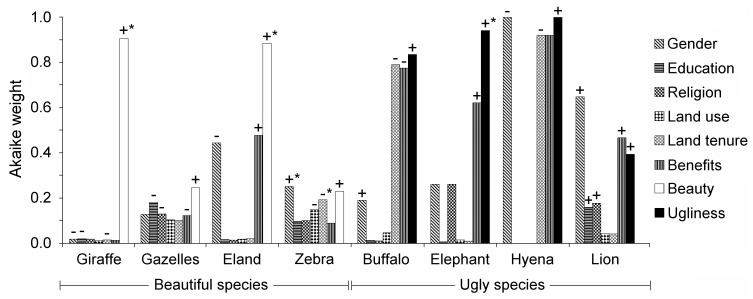
Relative importance of variables in most supported models explaining support for rescuing and removing species. (+) and (−) signs indicate a positive and negative relationship with the response variable in the most supported models (respectively, support for rescuing species and support for removing species); (*): 0.05 significance level.

We formally investigated the relationship between people perceiving giraffe, gazelles, eland and zebra as beautiful and supporting their rescue while controlling for personal and household socioeconomic variables. Insufficient responses regarding rescuing lion, elephant, wildebeest, ostrich, oryx and rhino precluded including them in this analysis. For the most supported models, the error distribution was modeled correctly and we did not detect departures from model assumptions. Beauty is the most important variable explaining support for rescuing giraffe, gazelles and eland, particularly for giraffe (W_i_ = 0.91) and eland (W_i_ = 0.88) (Figure 3; Tables S2, S3 and S4). The effects of beauty on support for rescuing gazelles and zebras are less pronounced, with other variables having comparable effects. For zebra (Table S5), in particular, their beauty (W_i_ = 0.23) is secondary when compared with the effects of being a woman (W_i_ = 0.25) on support for rescuing them. Being an agropastoralist and living on private land negatively influence support for rescuing zebra. Getting benefits from wildlife in the household only had an important positive effect in the case of eland (W_i_ = 0.48).

Of the variables tested for their influence on support for species’ removal, perceived ugliness was the most important, with the following Akaike weights: W_i_ = 0.83 (buffalo), W_i_ = 0.94 (elephant) and W_i_ = 0.99 (hyena) (Figure 3; Tables S6, S7 and S8, respectively). However, other variables than aesthetic appreciation also highly contribute to explaining support for removal of species. Notably, in the case of lion, being a woman (W_i_ = 0.64) and, surprisingly, the household getting economic benefits from wildlife (W_i_ = 0.47) more strongly explained support for removing them than perceiving them as ugly (Wi = 0.39). Economic benefits also, unexpectedly, influence support for elephant removal (W_i_ = 62). To a smaller extent, being a Christian and being formally educated explain support for lion removal.

## Discussion

### Influence of Human Aesthetic Appreciation on Attitudes towards Conservation

Our study highlights the influence of human aesthetic appreciation of wildlife species on attitudes towards their conservation in an African country’s rural area, the surroundings of southern Kenya’s Amboseli NP, a savanna ecosystem where wild mammals are highly visible. Perceiving a species as beautiful is strongly related to supporting its protection (“rescuing” it); and perceiving a species as ugly also explains support for its removal alongside other factors. Studies in industrialized countries have shown that human aesthetic appreciation of animal species influences public willingness to protect them and decisions about their conservation [10], [13], [14], particularly when it comes to targeting species for conservation efforts, across a range of taxa [2], [3], [9]. To our knowledge, our research is the first to formally explore and demonstrate the strength of this effect in an African rural setting by relating aesthetic appreciation of wildlife species to local attitudes towards their conservation.

As anticipated, the effects of perceived animal beauty and ugliness on attitudes towards species’ conservation varied in strength across species. Beauty is the strongest variable predisposing local residents to rescue giraffe, gazelles and eland relatively to personal and household explanatory variables. Although zebras were the species most often listed for their beauty, support for rescuing them is more strongly influenced by being a woman than by perceiving them as beautiful. This, however, is not a surprising result and might be indirectly related to aesthetics since women play a crucial role in Maasai culture in that regard: it is female artists who uphold the Maasai aesthetic codes of color contrast, complementarity and balance in their beadwork [54]. Zebra’s (*oloitiko*) coat most dramatically embodies these principles and there is a beadwork pattern named after it (*enkoitiko*) [69]. Agropastoralism and private land tenure negatively affected support for rescuing zebra. This is explained by the fact that zebra destroy crops and compete for grazing with cattle – problems that are more acutely felt on private ranches with rainfed cultivation, like in Osilalei. Contrary to previous studies’ findings [36], this land use effect was not found for the other species, including elephants [21].

Perceived ugliness was the most important variable influencing support for removal of buffalo, elephant and hyena. Interestingly, the effect of lion ugliness is less pronounced. This possibly reflects lions’ central and positive role in Maasai culture, in which they embody qualities that Maasai also admire in humans [26], [28], including aesthetic ones. This is especially so among Maasai *ilmurran* (“warriors”) who measure themselves against lion, the only animal they consider a worthy adversary [26].

As in other studies of drivers of attitudes towards wildlife, other variables were found to be influential. Being a female was the strongest factor positively influencing support for lion removal. This finding is in line with studies in the region [37], [39] and elsewhere [70], [71] that demonstrate the negative effect of being a woman on attitudes towards predators. Maasai women’s negative attitudes could be related to their feeling more fear of dangerous animals, such as lion, than men. Women’s fear of lions possibly results from their lesser exposure to them (see [70]). Men, instead, frequently confront predators in defense of their families and livestock [26]. This is corroborated by studies in the Serengeti [39] and in Europe [70], [72], where women’s negative attitudes towards predators are linked to fear (but see [47] and [26] for Amboseli narratives of lions protecting Maasai women and children and women being unafraid of lions). As in Hazzah et al. [40], being a Christian (and being formally educated) also positively influenced support for lion removal. Our qualitative analysis suggests that Evangelical and/or educated Maasai, who describe themselves as “modern”, tend not to support maintaining traditions like the *ilmurran* lion hunt – a practice associated with respecting and liking lions [26]. Like Gadd [21] and Groom and Harris [38], we found no effect of formal education on support for rescuing and for removing the other species.

Perceived dangerousness of animals, in turn, might shape perceptions of ugliness. One informant wondered about the link between his being scared of some animals and not enjoying looking at them. Several female informants also mentioned fear as an ugliness criterion. Thus, for animals whose perceived ugliness explains negatives attitudes towards their conservation, it is difficult to determine that this is strictly because of their *physical* unattractiveness or because of the fear these animals inspire. This overlap between perceptions of fear and perceptions of ugliness has conservation implications, as we discuss below.

Economic benefits from wildlife in the household, an important conservation tool in the study area, do not have clear effects on attitudes towards species’ conservation. In particular, we found small, nonexistent and even negative effects (in the case of gazelles) on support for rescuing species; and a surprisingly positive influence on supporting lion and elephant removal. The fact that, around Amboseli NP, wildlife’s monetary value is still mostly an alien concept could explain these unexpected results. Most households have not benefited economically from conservation because of inequitable distribution of revenues [38] and many informants confused the benefits’ sources: for instance, some ascribed benefits from conservation organizations to Christian ones (see also [21]). Our findings also suggest that providing monetary incentives might not be enough to curb negative attitudes towards the conservation of certain species when local perceptions of their ugliness and/or dangerousness are deep-seated sentiments, especially in contexts of insufficient knowledge about conservation benefits [21], [47] and antagonistic relationships with park authorities [28]. Economic incentive approaches to conservation have proved problematic elsewhere in East Africa (e.g., [73]). Studies have suggested that non-utilitarian, non-economic dimensions of human-wildlife coexistence around African protected areas should inform conservation strategy design [27], [74]. In Maasailand, an animal’s ability to “please the eye” is, so far, separate from its being perceived as economically profitable. However, as tourism and conservation emphasize wildlife’s economic value, we would hypothesize that creating expectations of economic gain from wildlife could compromise such local non-economic reasons for which wild animals are tolerated and even liked by the people coexisting with them if that profit does not materialize (see also [21]). Instead, conservation science should recognize that wild animals can be locally valued in non-utilitarian ways, even among natural resource-dependent communities, and strive to incorporate these dimensions in conservation strategy design.

### Aesthetics and Direct Exposure to Wild Animals

Besides the visual delight that wildlife offer them, Maasai also value the educational importance of *seeing* animals: “Wild animals are beautiful to look at and children can learn to differentiate between the harmless ones and the aggressive ones” (Elder, Imbirikani). For another Imbirikani elder “It would be good to have rhinos around because that would avoid taking children to Nairobi [National Park] to see them.” This, in turn, makes people value the presence of wildlife on their land, a sentiment expressed around other East African protected areas [27]. However, Stokes [5] claims that the power of an animal’s beauty as a motivator for its conservation does not exist for people who have not seen that animal. We voice the same concern for the Amboseli Ecosystem where direct exposure to some species has decreased (viewing them through audiovisual media is not an option). Land privatization in the ecosystem has displaced some species [55] and, indeed, interviewed Osilalei youth had never seen elephants, lions and buffaloes. Protected area delimitation has also curtailed exposure to wildlife: since Amboseli NP’s creation (1974), human settlement and herding within it are prohibited (except in droughts). Apart from those living at the park’s edge, few informants (especially from the more distant and privatized Osilalei area) had ever visited the park where species like lion, elephant and buffalo are more easily viewed. Herding is also restricted within the smaller conservation areas around the park in consideration of tourists’ aesthetic preferences (i.e., no cattle in “wilderness areas”). Socioeconomic changes (i.e., schooling; urban employment) also mean that Maasai youth spend less time observing wildlife while herding. These combined processes result in less frequent encounters with certain species and a concomitant loss in the knowledge people have about these species [75] and related aesthetic appreciation. It is ironic that many young people in Amboseli have not seen wild animals that are familiar, at least on paper or screen, to Westerners.

A strategy to counteract this downward trend in exposure to wild animals and improve local attitudes towards unpopular, “ugly” animals could involve providing opportunities for residents around East African protected areas to visit them (as most lack the means to do so) and be exposed to species they less frequently see. In Amboseli, this approach would be most beneficial with those “ugly” species that harm livelihoods and/or cause fear, i.e., buffaloes, elephants, hyenas and lions. Around the park, these animals are recurrently encountered while they are feeding on people’s crops or “harass[ing] cows”. Local explanations, however, suggest that people would enjoy watching them in less threatening contexts: “I like the elephant because I enjoy seeing it if it’s not eating crops. It's the biggest animal and I like seeing the biggest animals all the time” (Elder, Imbirikani). We also showed that species viewed as “ugly” by some informants have strong “eye pleasing” behavioral or physical characteristics to other informants, who consider them worth conserving for that reason. This suggests that “ugly” species could, over time, become “beautiful” if people have a chance to become more familiar with their interesting visual characteristics.

Heberlein [76] argues that while environmental attitudes are extremely difficult to change, especially through education, they do change as people have *direct experience,* which has been shown to raise public support for conservation of unpopular animals [70], [77], [78]. Depicting disliked species in an attractive manner can also improve public perceptions thereof [79], [80]. We thus hypothesize that making it easier for local residents in developing countries to safely and directly enjoy the sight of animals inside protected areas could contribute to offset negative attitudes resulting from perceptions of animal “ugliness” (physical unattractiveness and/or dangerousness), or a combination thereof. While some pioneering programs in Tanzania are promoting protected area visitation by local residents of both genders and all ages (see [81]) and the Kenya Wildlife Service runs education centers in national parks (although not in Amboseli NP) [42], more should be done (e.g., adults are excluded from school visits to Amboseli NP).

Heberlein [76] also reminds us that attitudes towards conservation do not necessarily translate into behaviors and that “settings and factors outside the individual have far more influence on what people do than beliefs, knowledge, or emotion – the drivers of attitudes” [76: 583]. The Amboseli Ecosystem illustrates this: conflicts between the park authorities and local communities have sparked political killings of lions, buffaloes and elephants by *ilmurran* in August 2012 [82]; and elephant poaching for ivory is rising [83]. Clearly, perceiving these species as beautiful is irrelevant in this context. Stern [29] argues that trust is the most critical aspect for building positive park-communities relationships. Facilitating visits to protected areas by local residents, as a display of goodwill by park management, could help build trust and ameliorate park-communities relationships where these are strained by local perceptions that conservationists and governments care more about wildlife than about human wellbeing (see also [84]), as is the case around Amboseli NP [47].

Finally, this widely implementable approach could complement economic incentive approaches to conservation and help overcome limitations of the human-wildlife conflict framework, which conceptualizes people and wildlife as antagonists [85]. Human attitudes towards wildlife are more complex and fluid than this framework presupposes [25], [26], [28]. Ethnographic work like this one can disclose such nuances and inform conservation strategy design [58] by showing how existing positive dimensions of human-wildlife relationships can be built upon. Bhola et al. [86] and Boone and Hobbs [87] advocate promoting wildlife mobility outside of East African protected areas. Bringing local residents into parks could be another step towards reestablishing some connection between people and wildlife where it has been negatively affected by a range of political economic factors, including protected area creation.

### Suggestions for Future Research

We suggest three directions for future research to complement our findings on the influence of human aesthetic appreciation on attitudes towards species’ conservation. A future study could compare local aesthetic perceptions of wildlife species before and after visits to protected areas, testing the hypothesis that exposure to those species improves both aesthetic judgments thereof and attitudes towards their conservation. Another research effort should explore in more depth the influence of perceived beauty of lions and elephants, the targets of important conservation efforts across the region. Limited free listing of these species as “to be rescued” prevented statistically evaluating how their attractiveness influences people’s support for their protection. Finally, promoting positive attitudes towards wild animals through exposure to them will work best with highly visible and charismatic savanna species. A future study should explore the importance of people’s aesthetic appreciation of wildlife in other biomes where animals are not as visible, such as forests, and test this approach’s feasibility.

Although specific aesthetic preferences for animal species may vary cross-culturally, appreciating beauty in nature is likely a universal sentiment, and a powerful one. We show that aesthetic appreciation of biodiversity is not restricted to the industrialized world by highlighting the diversity and significance of aesthetic judgments regarding wildlife among Kenya Maasai pastoralists and agropastoralists. We hope that this study stimulates the further exploration of aesthetic appreciation of wild animals among the many human communities around the world that live with or near them, and we recommend that this dimension be considered in both research on human-wildlife coexistence and conservation strategy design in developing countries.

## Supporting Information

Table S1 Demographic and socioeconomic attributes of the informants and their households. Numbers in parentheses are percentages.
(DOCX)

Table S2 Summary of all tested models of support for rescuing giraffe, gazelle, eland and zebra. AIC is Akaike’s Information Criterion; ΔAIC is AIC_i_ -minAIC; W_i_ is Akaike weight.
(DOCX)

Table S3 Summary of all tested models of support for rescuing gazelles. AIC is Akaike’s Information Criterion; ΔAIC is AIC_i_ -minAIC; W_i_ is Akaike weight.
(DOCX)

Table S4 Summary of all tested models of support for rescuing eland. AIC is Akaike’s Information Criterion; ΔAIC is AIC_i_ -minAIC; W_i_ is Akaike weight.
(DOCX)

Table S5 Summary of all tested models of support for rescuing zebra. AIC is Akaike’s Information Criterion; ΔAIC is AIC_i_ -minAIC; W_i_ is Akaike weight.
(DOCX)

Table S6 Summary of all tested models of support for removal of buffalo. AIC is Akaike’s Information Criterion; ΔAIC is AIC_i_ -minAIC; Wi is Akaike weight.
(DOCX)

Table S7 Summary of all tested models of support for removal of elephant. AIC is Akaike’s Information Criterion; ΔAIC is AIC_i_ -minAIC; Wi is Akaike weight.
(DOCX)

Table S8 Summary of all tested models of support for removal of hyena. AIC is Akaike’s Information Criterion; ΔAIC is AIC_i_ -minAIC; Wi is Akaike weight.
(DOCX)

Table S9 Summary of all tested models of support for removal of lion. AIC is Akaike’s Information Criterion; ΔAIC is AIC_i_ -minAIC; Wi is Akaike weight.
(DOCX)

Dataset S1 (XLSX)
