# Supplementary material for: Influence of Aesthetic Appreciation of Wildlife Species on Attitudes towards Their Conservation in Kenyan Agropastoralist Communities
Source: PLoS One. 2014 Feb 14;9(2):e88842. doi: 10.1371/journal.pone.0088842 (PMC3925186; doi:10.1371/journal.pone.0088842)
Supplement: Table S1 — Demographic and socioeconomic attributes of the informants and their households. Numbers in parentheses are percentages. (DOCX) [file pone.0088842.s001.docx]

Table S1. Demographic and socioeconomic attributes of the informants and their households. Numbers in parentheses are percentages.

| **Study area** | **Imbirikani (n=32))** | **Emeshenani (n=33)** | **Osilalei (n=31)** |
| --- | --- | --- | --- |
| **Household attributes** |  |  |  |
| Average household size | 6.69 | 12.88 | 7.13 |
| **Land use/economic activities** |  |  |  |
| Livestock only | 1 (3.1) | 10 (30.3) | 2 (6.5) |
| Livestock + cultivation | 0 (0.0) | 3 (9.1) | 10 (32.3) |
| Livestock + other^1^ | 3 (9.4) | 8 (24.2) | 2 (6.5) |
| Livestock + cultivation + other^1^ | 28 (87.5) | 12 (36.4) | 17 (54.8) |
| **Informant attributes** | **Imbirikani (n=64)** | **Emeshenani (n=65)** | **Osilalei (n=62)** |
| **Gender** |  |  |  |
| Women (n=82) | 29 (45.3) | 27 (41.5) | 26 (41.9) |
| Men (n=109) | 35 (54.7) | 38 (58.5) | 36 (58.1) |
| **Age/gender categories** |  |  |  |
| Girl (n=5) | 2 (3.1) | 1 (1.5) | 2 (3.2) |
| Boy (n=8) | 1 (1.6) | 1 (1.5) | 6 (9.7) |
| Woman (n=76) | 27 (42.2) | 26 (40.0) | 23 (37.1) |
| *Olmurrani* (n=11)^2^ | 4 (6.3) | 4 (6.2) | 3 (4.8) |
| Elder (n=91) | 30 (46.9) | 33 (50.8) | 28 (45.2) |
| **Formally educated informants** | 14 (21.9) | 3 (4.6) | 11 (17.7) |
| **Religious affiliation** |  |  |  |
| Christians (n=116) | 51 (79.7) | 31 (47.7) | 34 (54.8) |
| Traditional Maasai (n=75) | 13 (20.3) | 34 (52.3) | 28 (45.2) |

^1^ Other: businesses, employment.

^2^ Plural of *Ilmurran*.
