# Supplementary material for: Influence of Aesthetic Appreciation of Wildlife Species on Attitudes towards Their Conservation in Kenyan Agropastoralist Communities
Source: PLoS One. 2014 Feb 14;9(2):e88842. doi: 10.1371/journal.pone.0088842 (PMC3925186; doi:10.1371/journal.pone.0088842)
Supplement: Table S2 — Summary of all tested models of support for rescuing giraffe, gazelle, eland and zebra. AIC is Akaike’s Information Criterion; ΔAIC is AICi -minAIC; Wi is Akaike weight. (DOCX) [file pone.0088842.s002.docx]

**Table S2.** Summary of all tested models for support for rescuing giraffe, gazelle, eland and zebra. AIC is Akaike’s Information Criterion; ΔAIC is AIC_i_ -minAIC; W_i_ is Akaike weight.

| **GIRAFFE** | **AIC** | **ΔAIC** | **W_i_** | **Overdispersion** |
| --- | --- | --- | --- | --- |
| **Aesthetic judgment of species** |  |  |  |  |
| Beautiful | 229.6 | 0 | 0.409 | 1.30 |
| **Personal attributes** |  |  |  |  |
| Gender | 237.3 | 7.7 | 0.009 | 1.34 |
| Education | 237.3 | 7.7 | 0.009 | 1.34 |
| Religion | 237.4 | 7.8 | 0.008 | 1.35 |
| Gender + Education | 238.8 | 9.2 | 0.004 | 1.34 |
| Gender + Religion | 238.8 | 9.2 | 0.004 | 1.34 |
| Education + Religion | 238.7 | 9.1 | 0.004 | 1.34 |
| Gender + Education + Religion | 239.7 | 10.1 | 0.003 | 1.34 |
| **Household socioeconomic attributes** |  |  |  |  |
| Land use | 237.7 | 8.1 | 0.007 | 1.35 |
| Land tenure | 237.6 | 8 | 0.007 | 1.35 |
| Economic benefits from wildlife (Benefits) | 237.7 | 8.1 | 0.007 | 1.35 |
| Land use + Land tenure | 239.6 | 10 | 0.003 | 1.35 |
| Land use + Benefits | 239.6 | 10 | 0.003 | 1.35 |
| Land tenure + Benefits | 239.6 | 10 | 0.003 | 1.35 |
| Land use + Benefits+ Land tenure | 241.6 | 12 | 0.001 | 1.35 |
| **Personal + Household socioeconomic attributes** |  |  |  |  |
| Education + Land tenure | 239.3 | 9.7 | 0.003 | 1.34 |
| **Personal attributes + Aesthetic judgment** |  |  |  |  |
| Education + Beautiful | 230.6 | 1 | 0.248 | 1.40 |
| **Household socioeconomic attributes + Aesthetic judgment** |  |  |  |  |
| Land tenure + Beautiful | 231.5 | 1.9 | 0.158 | 1.30 |
| **Personal + Household socioeconomic attributes + Aesthetic judgment** |  |  |  |  |
| Education + Land tenure + Beautiful | 232.6 | 3 | 0.091 | 1.29 |
| Null | 235.7 | 6.1 | 0.019 |  |
