# Supplementary material for: Influence of Aesthetic Appreciation of Wildlife Species on Attitudes towards Their Conservation in Kenyan Agropastoralist Communities
Source: PLoS One. 2014 Feb 14;9(2):e88842. doi: 10.1371/journal.pone.0088842 (PMC3925186; doi:10.1371/journal.pone.0088842)
Supplement: Table S3 — Summary of all tested models of support for rescuing gazelles. AIC is Akaike’s Information Criterion; ΔAIC is AICi -minAIC; Wi is Akaike weight. (DOCX) [file pone.0088842.s003.docx]

| **GAZELLES** | **AIC** | **ΔAIC** | **Wi** | **Overdispersion** |
| --- | --- | --- | --- | --- |
| **Aesthetic judgment of species** |  |  |  |  |
| Beautiful | 264.2 | 0.0 | 0.124 | 1.36 |
| **Personal attributes** |  |  |  |  |
| Gender | 266.4 | 2.2 | 0.041 | 1.37 |
| Education | 265.1 | 0.9 | 0.079 | 1.40 |
| Religion | 265.9 | 1.7 | 0.053 | 1.37 |
| Gender + Education | 266.2 | 2.0 | 0.046 | 1.37 |
| Gender + Religion | 267.7 | 3.5 | 0.022 | 1.37 |
| Education + Religion | 266.6 | 2.4 | 0.037 | 1.37 |
| Gender + Education + Religion | 268.0 | 3.8 | 0.019 | 1.37 |
| **Household socioeconomic attributes** |  |  |  |  |
| Land use | 266.3 | 2.1 | 0.043 | 1.37 |
| Land tenure | 266.6 | 2.4 | 0.037 | 1.37 |
| Benefits | 266.0 | 1.8 | 0.050 | 1.37 |
| Land use + Land tenure | 268.0 | 3.8 | 0.019 | 1.37 |
| Land use + Benefits | 267.3 | 3.1 | 0.026 | 1.36 |
| Land tenure + Benefits | 267.0 | 2.8 | 0.031 | 1.36 |
| Land use + Land tenure + Benefits | 268.4 | 4.2 | 0.015 | 1.36 |
| **Aesthetic judgment** |  |  |  |  |
| Beautiful | 264.2 | 0.0 | 0.124 | 1.36 |
| **Personal + Household socioeconomic attributes** |  |  |  |  |
| Education + Benefits | 266.3 | 2.1 | 0.043 | 1.36 |
| **Personal + Aesthetic judgment** |  |  |  |  |
| Education + Beautiful | 264.5 | 0.3 | 0.107 | 1.35 |
| **Household socioeconomic attributes + Aesthetic judgment** |  |  |  |  |
| Benefits + Beautiful | 265.5 | 1.3 | 0.065 | 1.36 |
| **Personal + Household socioeconomic attributes + Aesthetic judgment** |  |  |  |  |
| Education + Benefits + Beautiful | 265.8 | 1.6 | 0.056 | 1.35 |
| Null | 264.9 | 0.7 | 0.087 |  |

**Table S3.** Summary of all tested models for support for rescuing gazelles. AIC is Akaike’s Information Criterion; ΔAIC is AIC_i_ -minAIC; W_i_ is Akaike weight.
