# Supplementary material for: Influence of Aesthetic Appreciation of Wildlife Species on Attitudes towards Their Conservation in Kenyan Agropastoralist Communities
Source: PLoS One. 2014 Feb 14;9(2):e88842. doi: 10.1371/journal.pone.0088842 (PMC3925186; doi:10.1371/journal.pone.0088842)
Supplement: Table S4 — Summary of all tested models of support for rescuing eland. AIC is Akaike’s Information Criterion; ΔAIC is AICi -minAIC; Wi is Akaike weight. (DOCX) [file pone.0088842.s004.docx]

| **ELAND** | **AIC** | **ΔAIC** | **Wi** | **Overdispersion** |
| --- | --- | --- | --- | --- |
| **Aesthetic judgment of species** |  |  |  |  |
| Beautiful | 157.3 | 0.1 | 0.023 | 1.26 |
| **Personal attributes** |  |  |  |  |
| Gender | 162.9 | 5.7 | 1.318 | 1.36 |
| Education | 166.4 | 9.2 | 2.128 | 1.39 |
| Religion | 166.2 | 9.0 | 2.082 | 1.39 |
| Education + Gender | 164.0 | 6.8 | 1.573 | 1.38 |
| Education + Religion | 168.0 | 10.8 | 2.498 | 1.42 |
| Religion + Gender | 164.8 | 7.6 | 1.758 | 1.38 |
| Religion + Gender + Education | 166.0 | 8.8 | 2.035 | 1.39 |
| **Household socioeconomic attributes** |  |  |  |  |
| Land use | 166.6 | 9.4 | 0.002 | 1.34 |
| Land tenure | 166.6 | 9.4 | 0.002 | 1.34 |
| Benefits | 161.6 | 4.4 | 0.027 | 1.32 |
| Land use + Land tenure | 168.6 | 11.4 | 0.001 | 1.34 |
| Land use +Benefits | 163.5 | 6.3 | 0.010 | 1.30 |
| Land tenure + Benefits | 163.1 | 5.9 | 0.013 | 1.29 |
| Land use + Benefits + Land tenure | 165.0 | 7.8 | 0.005 | 1.29 |
| **Personal + Household socioeconomic attributes** |  |  |  |  |
| Gender + Benefits | 163 | 5.8 | 0.013 | 1.28 |
| **Personal attributes + Aesthetic judgment** |  |  |  |  |
| Gender + Beautiful | 157.4 | 0.1 | 0.023 | 1.31 |
| **Household socioeconomic attributes + Aesthetic judgment** |  |  |  |  |
| Benefits + Beautiful | 157.2 | 0 | 0.243 | 1.26 |
| **Personal + Household socioeconomic attributes + Aesthetic judgment** |  |  |  |  |
| Gender + Benefits + Beautiful | 157.7 | 0.5 | 0.189 | 1.23 |
| Null | 164.7 | 7.5 | 0.006 |  |

**Table S4.** Summary of all tested models for support for rescuing eland. AIC is Akaike’s Information Criterion; ΔAIC is AIC_i_ -minAIC; W_i_ is Akaike weight.
