# Supplementary material for: Influence of Aesthetic Appreciation of Wildlife Species on Attitudes towards Their Conservation in Kenyan Agropastoralist Communities
Source: PLoS One. 2014 Feb 14;9(2):e88842. doi: 10.1371/journal.pone.0088842 (PMC3925186; doi:10.1371/journal.pone.0088842)
Supplement: Table S5 — Summary of all tested models of support for rescuing zebra. AIC is Akaike’s Information Criterion; ΔAIC is AICi -minAIC; Wi is Akaike weight. (DOCX) [file pone.0088842.s005.docx]

| **ZEBRA** | **AIC** | **ΔAIC** | **Wi** | **Overdispersion** |
| --- | --- | --- | --- | --- |
| **Aesthetic judgment of species** |  |  |  |  |
| Beautiful | 190.8 | 3.7 | 0.610 | 1.36 |
| **Personal attributes** |  |  |  |  |
| Gender | 187.6 | 0.5 | 0.082 | 1.34 |
| Education | 191.5 | 4.4 | 0.725 | 1.37 |
| Christian | 191.6 | 4.5 | 0.741 | 1.37 |
| Gender + Education | 189.6 | 2.5 | 0.412 | 1.35 |
| Gender + Religion | 189.5 | 2.4 | 0.395 | 1.35 |
| Education + Religion | 193.5 | 6.4 | 1.054 | 1.38 |
| Gender + Education + Religion | 190.7 | 3.6 | 0.593 | 1.36 |
| **Household socioeconomic attributes** |  |  |  |  |
| Land use | 189.9 | 2.8 | 0.461 | 1.36 |
| Land tenure | 188.8 | 1.7 | 0.280 | 1.35 |
| Benefits | 191.6 | 4.5 | 0.741 | 1.37 |
| Land use + Land tenure | 189.0 | 1.9 | 0.313 | 1.35 |
| Land use + Benefits | 191.9 | 4.8 | 0.791 | 1.37 |
| Land tenure + Benefits | 190.5 | 3.4 | 0.560 | 1.36 |
| Land use + Land tenure + Benefits | 190.7 | 3.6 | 0.593 | 1.36 |
| **Personal + Household socioeconomic attributes** |  |  |  |  |
| Gender + Land tenure | 187.1 | 0.0 | 0.000 | 1.34 |
| **Personal attributes + Aesthetic judgment** |  |  |  |  |
| Gender + Beautiful | 188.6 | 1.5 | 0.247 | 1.35 |
| **Household socioeconomic attributes + Aesthetic judgment** |  |  |  |  |
| Land tenure + Beautiful | 190.2 | 3.1 | 0.511 | 1.36 |
| **Personal + Household socioeconomic attributes + Aesthetic judgment** |  |  |  |  |
| Gender + Land tenure + Beautiful | 188.3 | 1.2 | 0.198 | 1.35 |
| Null | 189.6 | 2.5 | 0.287 | 0.05 |

**Table S5.** Summary of all tested models for support for rescuing zebra. AIC is Akaike’s Information Criterion; ΔAIC is AIC_i_ -minAIC; W_i_ is Akaike weight.
