# Supplementary material for: Influence of Aesthetic Appreciation of Wildlife Species on Attitudes towards Their Conservation in Kenyan Agropastoralist Communities
Source: PLoS One. 2014 Feb 14;9(2):e88842. doi: 10.1371/journal.pone.0088842 (PMC3925186; doi:10.1371/journal.pone.0088842)
Supplement: Table S6 — Summary of all tested models of support for removal of buffalo. AIC is Akaike’s Information Criterion; ΔAIC is AICi -minAIC; Wi is Akaike weight. (DOCX) [file pone.0088842.s006.docx]

**Table S6.** Summary of all tested models for support for removal of buffalo. AIC is Akaike’s Information Criterion; ΔAIC is AIC_i_ -minAIC; Wi is Akaike weight.

| **BUFFALO** | **AIC** | **ΔAIC** | **Wi** | **Overdispersion** |
| --- | --- | --- | --- | --- |
| **Aesthetic judgment of species** |  |  |  |  |
| Ugly | 177.6 | 4.3 | 0.028 | 1.281 |
| **Personal attributes** |  |  |  |  |
| Gender | 178.8 | 5.5 | 0.015 | 1.290 |
| Education | 183.1 | 9.8 | 0.002 | 1.322 |
| Religion | 183.4 | 10.1 | 0.002 | 1.324 |
| Gender + Education | 180.7 | 7.4 | 0.006 | 1.289 |
| Gender + Religion | 180.7 | 7.4 | 0.006 | 1.289 |
| Education + Religion | 184.9 | 11.6 | 0.001 | 1.320 |
| Gender + Education + Religion | 182.7 | 9.4 | 0.002 | 1.289 |
| **Household socioeconomic attributes** |  |  |  |  |
| Land use | 181.9 | 8.6 | 0.003 | 1.313 |
| Land tenure | 179.5 | 6.2 | 0.011 | 1.295 |
| Benefit | 181.3 | 8.0 | 0.004 | 1.308 |
| Land use + Land tenure | 179.8 | 6.5 | 0.009 | 1.282 |
| Land use + Benefits | 178.2 | 4.9 | 0.021 | 1.270 |
| Land tenure +Benefits | 178.2 | 4.9 | 0.021 | 1.270 |
| Land use + Land tenure + Benefits | 179.2 | 5.9 | 0.012 | 1.263 |
| **Personal + Household socioeconomic attributes** |  |  |  |  |
| Gender + Land tenure + Benefits | 176.8 | 4.8 | 0.041 | 1.245 |
| **Personal attributes + Aesthetic judgment** |  |  |  |  |
| Gender + Ugly | 174.8 | 1.5 | 0.183 | 1.245 |
| **Household socioeconomic attributes + Aesthetic judgment** |  |  |  |  |
| Land tenure + Benefits + Ugly | 173.3 | 1.3 | 0.239 | 1.219 |
| **Personal + Household socioeconomic attributes + Aesthetic judgment** |  |  |  |  |
| Gender + Land tenure + Benefits + Ugly | 172 | 0.0 | 0.457 | 1.194 |
| Null | 181.5 | 8.2 | 0.006 | 1.325 |
