# Supplementary material for: Influence of Aesthetic Appreciation of Wildlife Species on Attitudes towards Their Conservation in Kenyan Agropastoralist Communities
Source: PLoS One. 2014 Feb 14;9(2):e88842. doi: 10.1371/journal.pone.0088842 (PMC3925186; doi:10.1371/journal.pone.0088842)
Supplement: Table S7 — Summary of all tested models of support for removal of elephant. AIC is Akaike’s Information Criterion; ΔAIC is AICi -minAIC; Wi is Akaike weight. (DOCX) [file pone.0088842.s007.docx]

| **ELEPHANT** | **AIC** | **ΔAIC** | **Wi** | **Overdispersion** |
| --- | --- | --- | --- | --- |
| **Aesthetic judgment of species** |  |  |  |  |
| Ugly | 242.6 | 1.1 | 0.255 | 1.286 |
| **Personal attributes** |  |  |  |  |
| Gender | 251.5 | 10 | 0.003 | 1.334 |
| Education | 251.6 | 10.1 | 0.003 | 1.335 |
| Religion | 251.1 | 9.6 | 0.004 | 1.332 |
| Gender + Education | 253 | 11.5 | 0.001 | 1.332 |
| Gender + Religion | 251.4 | 9.9 | 0.003 | 1.323 |
| Education + Religion | 252.7 | 11.2 | 0.002 | 1.330 |
| Gender + Education + Religion | 253.3 | 11.8 | 0.001 | 1.322 |
| **Household socioeconomic attributes** |  |  |  |  |
| Land use | 250.7 | 9.2 | 0.004 | 1.330 |
| Land tenure | 252.4 | 10.9 | 0.002 | 1.339 |
| Benefit | 249.4 | 7.9 | 0.009 | 1.323 |
| Land use + Land tenure | 252.7 | 11.2 | 0.002 | 1.330 |
| Land use + Benefits | 250 | 8.5 | 0.006 | 1.315 |
| Land tenure + Benefits | 251.2 | 9.7 | 0.003 | 1.322 |
| Land use + Land tenure + Benefits | 251.9 | 10.4 | 0.002 | 1.315 |
| **Personal + Household socioeconomic attributes** |  |  |  |  |
| Gender + Christian + Benefits | 250.5 | 9 | 0.005 | 1.307 |
| **Personal attributes + Aesthetic judgment** |  |  |  |  |
| Gender + Christian + Ugly | 244.6 | 3.1 | 0.094 | 1.275 |
| **Household socioeconomic attributes + Aesthetic judgment** |  |  |  |  |
| Benefits + Ugly | 241.5 | 0 | 0.442 | 1.269 |
| **Personal + Household socioeconomic attributes + Aesthetic judgment** |  |  |  |  |
| Gender + Religion + Benefits + Ugly | 243.6 | 2.1 | 0.155 | 1.280 |
| Null | 250.4 | 8.9 | 0.005 | 1.339 |

**Table S7.** Summary of all tested models for support for removal of elephant. AIC is Akaike’s Information Criterion; ΔAIC is AIC_i_ -minAIC; Wi is Akaike weight.
