# Supplementary material for: Influence of Aesthetic Appreciation of Wildlife Species on Attitudes towards Their Conservation in Kenyan Agropastoralist Communities
Source: PLoS One. 2014 Feb 14;9(2):e88842. doi: 10.1371/journal.pone.0088842 (PMC3925186; doi:10.1371/journal.pone.0088842)
Supplement: Table S8 — Summary of all tested models of support for removal of hyena. AIC is Akaike’s Information Criterion; ΔAIC is AICi -minAIC; Wi is Akaike weight. (DOCX) [file pone.0088842.s008.docx]

| **HYENA** | **AIC** | **ΔAIC** | **Wi** | **Overdispersion** |
| --- | --- | --- | --- | --- |
| **Personal attributes** |  |  |  |  |
| Gender | 214.1 | 23.6 | 0.000 | 1.131 |
| Education | 248.5 | 58 | 0.000 | 1.318 |
| Religion | 245.2 | 54.7 | 0.000 | 1.300 |
| Education + Gender | 215.3 | 24.8 | 0.000 | 1.127 |
| Education + Christian | 216 | 25.5 | 0.000 | 1.130 |
| Christian + Gender | 246.8 | 56.3 | 0.000 | 1.298 |
| Christian + Gender + Education | 217.3 | 26.8 | 0.000 | 1.127 |
| **Household socioeconomic attributes** |  |  |  |  |
| Land use | 248.4 | 57.9 | 0.000 | 1.317 |
| Land tenure | 240.3 | 49.8 | 0.000 | 1.273 |
| Benefits | 248.5 | 58 | 0.000 | 1.318 |
| Land use + Land tenure | 242 | 51.5 | 0.000 | 1.272 |
| Land use +Benefits | 250.4 | 59.9 | 0.000 | 1.317 |
| Land tenure + Benefits | 242.3 | 51.8 | 0.000 | 1.273 |
| Land use + Benefits + Land tenure | 243.9 | 53.4 | 0.000 | 1.271 |
| **Aesthetic judgment of species** |  |  |  |  |
| Ugly | 212 | 21.5 | 0.000 | 1.120 |
| **Personal + Household socioeconomic attributes** |  |  |  |  |
| Gender + Land tenure | 207.3 | 16.8 | 0.000 | 1.083 |
| **Personal attributes + Aesthetic judgment** |  |  |  |  |
| Gender + Ugly | 195.4 | 4.9 | 0.079 | 1.018 |
| **Household socioeconomic attributes + Aesthetic judgment** |  |  |  |  |
| Land tenure + Ugly | 208.6 | 18.1 | 0.000 | 1.090 |
| **Personal + Household socioeconomic attributes + Aesthetic judgment** |  |  |  |  |
| Gender + Land tenure + Ugly | 190.5 | 0 | 0.920 | 0.997 |
| Null | 246.5 | 56 | 0.000 | 1.318 |

**Table S8.** Summary of all tested models for support for removal of hyena. AIC is Akaike’s Information Criterion; ΔAIC is AIC_i_ -minAIC; Wi is Akaike weight.
