# Supplementary material for: Influence of Aesthetic Appreciation of Wildlife Species on Attitudes towards Their Conservation in Kenyan Agropastoralist Communities
Source: PLoS One. 2014 Feb 14;9(2):e88842. doi: 10.1371/journal.pone.0088842 (PMC3925186; doi:10.1371/journal.pone.0088842)
Supplement: Table S9 — Summary of all tested models of support for removal of lion. AIC is Akaike’s Information Criterion; ΔAIC is AICi -minAIC; Wi is Akaike weight. (DOCX) [file pone.0088842.s009.docx]

**Table S9.** Summary of all tested models for support for removal of lion. AIC is Akaike’s Information Criterion; ΔAIC is AIC_i_ -minAIC; Wi is Akaike weight.

| **LION** | **AIC** | **ΔAIC** | **Wi** | **Overdispersion** |
| --- | --- | --- | --- | --- |
| **Aesthetic judgment of species** |  |  |  |  |
| Ugly | 215.8 | 3.0 | 0.037 | 1.328 |
| **Personal attributes** |  |  |  |  |
| Gender | 214.0 | 1.2 | 0.090 | 1.316 |
| Education | 218.1 | 5.3 | 0.012 | 1.342 |
| Religion | 218.1 | 5.3 | 0.012 | 1.342 |
| Gender + Education | 215.0 | 2.2 | 0.055 | 1.310 |
| Gender + Religion | 214.4 | 1.6 | 0.074 | 1.306 |
| Education + Religion | 219.4 | 6.6 | 0.006 | 1.338 |
| Gender + Education + Religion | 214.1 | 1.3 | 0.086 | 1.292 |
| **Household socioeconomic attributes** |  |  |  |  |
| Land use | 218.4 | 5.6 | 0.010 | 1.344 |
| Land tenure | 218.3 | 5.5 | 0.011 | 1.344 |
| Economic benefits from wildlife (Benefits) | 215.1 | 2.3 | 0.052 | 1.323 |
| Land use + Land tenure | 220.0 | 7.2 | 0.004 | 1.342 |
| Land use + Benefits | 217.0 | 4.2 | 0.020 | 1.323 |
| Land tenure + Benefits | 217.1 | 4.3 | 0.019 | 1.323 |
| Land use + Benefit + Land tenure | 219.0 | 6.2 | 0.007 | 1.323 |
| **Personal + Household socioeconomic attributes** |  |  |  |  |
| Gender + Benefits | 213.4 | 0.6 | 0.122 | 1.300 |
| **Personal attributes + Aesthetic judgment** |  |  |  |  |
| Gender + Ugly | 213.6 | 0.8 | 0.110 | 1.301 |
| **Household socioeconomic attributes + Aesthetic judgment** |  |  |  |  |
| Benefits + Ugly | 214.2 | 1.4 | 0.082 | 1.305 |
| **Personal + Household socioeconomic attributes + Aesthetic judgment** |  |  |  |  |
| Gender + Benefits + Ugly | 212.8 | 0.0 | 0.165 | 1.284 |
| Null | 216.5 | 3.7 | 0.026 | 1.345 |
